# ADAM17 Promotes Motility, Invasion, and Sprouting of Lymphatic Endothelial Cells

**DOI:** 10.1371/journal.pone.0132661

**Published:** 2015-07-15

**Authors:** Renata Mężyk-Kopeć, Barbara Wyroba, Krystyna Stalińska, Tomasz Próchnicki, Karolina Wiatrowska, Witold W. Kilarski, Melody A. Swartz, Joanna Bereta

**Affiliations:** 1 Department of Cell Biochemistry, Faculty of Biochemistry, Biophysics and Biotechnology, Jagiellonian University in Kraków, Kraków, Poland; 2 Institute of Bioengineering and Swiss Institute for Cancer Research (ISREC), School of Life Sciences, École Polytechnique Fédérale de Lausanne (EPFL), Lausanne, Switzerland; 3 Institute for Molecular Engineering, University of Chicago, Chicago, Illinois, United States of America; University of Illinois at Chicago, UNITED STATES

## Abstract

Tumor-associated lymphatic vessels actively participate in tumor progression and dissemination. ADAM17, a sheddase for numerous growth factors, cytokines, receptors, and cell adhesion molecules, is believed to promote tumor development, facilitating both tumor cell proliferation and migration, as well as tumor angiogenesis. In this work we addressed the issue of whether ADAM17 may also promote tumor lymphangiogenesis. First, we found that ADAM17 is important for the migratory potential of immortalized human dermal lymphatic endothelial cells (LEC). When ADAM17 was stably silenced in LEC, their proliferation was not affected, but: (*i*) single-cell motility, (*ii*) cell migration through a 3D Matrigel/collagen type I matrix, and (*iii*) their ability to form sprouts in a 3D matrix were significantly diminished. The differences in the cell motility between ADAM17-proficient and ADAM17-silenced cells were eliminated by inhibitors of EGFR and HER2, indicating that ADAM17-mediated shedding of growth factors accounts for LEC migratory potential. Interestingly, ADAM17 depletion affected the integrin surface expression/functionality in LEC. ADAM17-silenced cells adhered to plastic, type I collagen, and fibronectin faster than their ADAM17-proficient counterparts. The difference in adhesion to fibronectin was abolished by a cyclic RGD peptide, emphasizing the involvement of integrins in the process. Using a soluble receptor array, we identified BIG-H3 among several candidate proteins involved in the phenotypic and behavioral changes of LEC upon ADAM17 silencing. In additional assays, we confirmed the increased expression of BIG-H3, as well as TGFβ2 in ADAM17-silenced LEC. The antilymphangiogenic effects of ADAM17 silencing in lymphatic endothelial cells suggest further relevance of ADAM17 as a potential target in cancer therapy.

## Introduction

Cancer is the leading cause of death in developed countries. Although localized tumors are successfully treated by surgery, chemo- and radiotherapy, as well as new, sophisticated methods, medicine is often helpless in the face of metastatic disease. Therefore, many efforts are being made to understand the molecular mechanisms underlying the processes that support tumor spreading to distant organs through blood and lymphatic vessels. The involvement of the lymphatic system in tumor metastasis has been generally accepted for a long time and the presence of cancer cells in lymph nodes is a major criterion in tumor staging [1]. In the last 20 years, ample evidence has emerged that correlates lymphangiogenesis in the tumor microenvironment with metastasis and poor prognosis; intensive research has led to the consistent, albeit still largely incomplete, picture of the relationship between the growing tumor and the lymphatic system. Malignant tumors, mostly due to the expression of pro-lymphangiogenic factors VEGF-C and VEGF-D, induce the expansion of lymphatic vessels in the tumor vicinity, as well as in the sentinel lymph nodes. The correlation between the levels of these growth factors and the density of intra- and peritumoral lymphatics, as well as the tumor cell incidence in draining lymph nodes and poor patient prognosis, are already well documented by preclinical and clinical studies [1]. The primary perception of lymphatic vessels simply as ducts for tumor cell dissemination changed dramatically in the light of recent discoveries of immunoregulatory functions of LEC. They are the source of chemokines such as CCL21, CCL27 and CCL1 [2–4], which attract immune cells and certain tumor cells to the lymph nodes (reviewed in [1]). LEC have also been shown to induce immune tolerance [5], at least partially due to their high expression of PD-L1 [6, 7]. Lymphangiogenesis is not limited to tumor development, but is also associated with inflammation. In both conditions, similar mechanisms and factors drive this complex process; LEC proliferation and migration, as well as remodeling of the extracellular matrix (ECM), are critically important [8, 9].

A disintegrin and metalloprotease 17 (ADAM17) is a sheddase, a protease that releases extracellular domains of transmembrane proteins, which thereby modulate cell-cell and cell-environment communication [10]. Among almost eighty ADAM17 substrates described to date, special attention is paid to growth factors and cytokines, their receptors, and cell adhesion molecules, which are regulators of vital functions such as growth, differentiation and immunity [11]. ADAM17 overexpression has been demonstrated in numerous human tumors (reviewed in [12]) and several well-designed studies have shown correlations between the levels of ADAM17 expression and tumor progression [13–16]. According to current views, the major mechanism by which ADAM17 supports cancer development involves shedding, and thus activation, of growth factors of the EGF family such as TGFα, HB-EGF, amphiregulin or neuregulins [17]. In turn, these growth factors stimulate survival, proliferation and migration of tumor cells, often in an autocrine manner [12, 18]. They may also contribute to tumor progression by inducing VEGF-A synthesis and thereby promoting tumor angiogenesis [13, 19, 20]. Indeed, it has been shown that ADAM17 may control endothelial cell proliferation, migration and tube formation, as well as the expression of matrix metalloproteinase-2, which is involved in basement membrane and ECM degradation during angiogenesis [19, 21]. Although the targeting of ADAM17 is considered a promising strategy in tumor therapy [22, 23], its role in tumor cell dissemination into lymphatic vessels, as well as its effect on lymphatic endothelial cells in general, has not yet been explored. While mice with ADAM17 knockout in endothelial cells have been generated, they have been used thus far to investigate the role of ADAM17 in physiology and pathology of vascular rather than of lymphatic endothelium [24].

Herein, we asked whether ADAM17 plays a role in lymphangiogenesis. Our results show that even though ADAM17 does not influence LEC proliferation, it has important roles in supporting LEC motility, migration, and invasion into a 3D matrix, processes central to lymphangiogenesis. The autocrine HB-EGF-EGFR/HER2 signaling initiated by ADAM17 seems to be of particular significance for LEC motility. Concomitantly, ADAM17 affects the status of integrins, important for cell adhesion. Silencing of ADAM17 alters the LEC transcriptome and proteome, including an increase in TGFβ2 and BIG-H3 expression. These findings suggest a new role for ADAM17 in lymphangiogenesis, and provide additional rationale for targeting ADAM17 in cancer.

## Materials and Methods

### Cell Culture

Human dermal lymphatic endothelial cell line (LEC) was a gift from Prof. Dontscho Kerjaschki (Medical University of Vienna, Austria). This cell line was derived from an hTERT-immortalized endothelial cell population originally containing blood endothelial and lymphatic endothelial cells [25, 26]. The LEC line retains lymphatic endothelial characteristics and functionality over at least 30 passages [27].

All cells were cultured in Endothelial Growth Medium (EGM2 BulletKit, Lonza) under standard conditions. The culture medium for ADAM17-silenced and mock-transduced sublines of LEC was supplemented with puromycin (0.5 μg/ml). The cells were passaged using Accutase (Promega). For most experiments, the cells were maintained in Endothelial Basal Medium (EBM, Lonza) supplemented with 2% fetal calf serum (FCS, Lonza), vitamin C and heparin, hereafter referred to as the basal medium. In some experiments, the cells were treated with 12.5 μM or 25 μM GM6001 (Calbiochem), a broad-spectrum metalloprotease inhibitor, or with 10 μM GW280264X (AOBIOUS), a specific inhibitor of ADAM10 and ADAM17, or incubated with DMSO (GM6001 and GW280264X solvent) at a corresponding concentration. Routine PCR testing for mycoplasma 16S rDNA confirmed the absence of contamination.

### Silencing of ADAM17 in LEC

LEC seeded on 12-well plates (4×10^3^ cells/well) were transduced with lentiviral vectors coding for (*i*) ADAM17 shRNA sequence no. 2 (TRCN0000294262), (*ii*) ADAM17 shRNA sequences nos. 3 and 5 (TRCN0000311680 and TRCN0000286913, respectively), or (*iii*) non-interfering control shRNA (Mission shRNA, Sigma-Aldrich) at MOI = 5, in the presence of polybrene (8 μg/ml). Stably transduced cell lines were obtained by puromycin selection (0.5 μg/ml). The efficiency of ADAM17 silencing was verified by real-time PCR (qRT-PCR) and western blotting analyses. The lack of an interferon response to the shRNAs was confirmed by quantifying the expression of interferon-inducible 2’-5’-oligoadenylate synthase 1 (OAS1) using qRT-PCR (below).

### Analysis of transcript levels by qRT-PCR

Total RNA was isolated from the cells by guanidinium thiocyanate-phenol-chloroform extraction and 500 ng of each sample was reverse-transcribed to cDNA using MMLV reverse transcriptase (Promega). cDNA samples (~10 ng) were amplified with One-Step qRT-PCR kit (Kapa Biosystems) using the Eco Real-Time PCR System (Illumina). The following primers (200 nM) were used:

ADAM17 –F: TGCAGTGACAGGAACAGTCC, R: GGATGCATTTCCCATCCTTA;

OAS1 –F: TTCTCCACCTGCTTCACAGA, R: TGGGCTGTGTTGAAATGTGT;

EGFR–F: AGTGACTGCTGCCACAACC, R: GGTGGCACCAAAGCTGTAATT

HER2 –F: ATAGACACCAACCGCTCTCG, R: ACAGATGCCACTGTGGTTGA

HER3 –F: GCCAATGAGTTCACCAGGAT, R: ACGTGGCCGATTAAGTGTTC

HER4 –F: ATGGCCTTCCAACATGACTC, R: CACCTGCCATCACATTGTTC

BIG-H3 –F: ATGCTTGAAGGTAACGGCCA, R: CGCCTTCCCGTTGATAGTGA;

TGFβ1 –F: AGTGGACATCAACGGGTTCAG, R: CATGAGAAGCAGGAAAGGCC;

TGFβ2 –F: CTGTCCCTGCTGCACTTTTGTA, R: TGTGGAGGTGCCATCAATACCT;

TGFβ3 –F: GGACTTCGGCCACATCAAGA, R: ATAGGGGACGTGGGTCATCA;

EF2 –F: CGAGATCAAGGACAGTGTGG, R: AAGGTAGATGGGCTCCATGA.

The levels of various mRNAs in ADAM17-silenced cell lines were compared to those in the mock-transduced and the wild-type cell lines using the delta delta Ct relative quantitation method. EF2 cDNA was used as reference.

### Western blotting

The cells of each LEC subline (3×10^5^) were lysed on ice in RIPA buffer containing 25 mM Tris-HCl pH 7.4, 150 mM NaCl, 0.1% SDS, 1% NP-40 and Complete Protease Inhibitor Cocktail (Roche Applied Science). Western blotting analysis was performed according to a standard protocol [28]. Membranes were probed with the following antibodies at the indicated dilutions: rabbit polyclonal anti-ADAM17 (Abcam), 1:5,000; mouse monoclonal anti-BIG-H3 (Proteintech), 1:1,000; mouse monoclonal anti HB-EGF (MyBioSource), 1.5 μg/ml; rabbit monoclonal anti-EGFR (EP38Y) (Abcam), 1:100,000; rabbit polyclonal anti-ErbB2 (Abcam), 1:1,000; and mouse monoclonal anti-β-actin (Biodesign Int.), 1:5,000. Appropriate HRP-conjugated secondary antibodies (Sigma-Aldrich) were used. Bands were visualized using Immobilon Western Chemiluminescent HRP Substrate (Millipore).

### ELISA

LEC sublines were plated in 6-cm-diameter plates (5×10^5^ cells/plate) and cultured overnight in complete medium. Next, the cells were incubated in basal medium (8 h) and finally in 2.5 ml of fresh basal medium (24 h). The levels of TGFβ1 and TGFβ2 in undiluted media were measured with ELISA kits for TGFβ1 (eBioscience) and for TGFβ2 (R&D Systems), according to the manufacturers’ protocols.

### Proliferation/viability assay

The cells of each LEC subline were plated in 24-well plates (5×10^3^ cells/well) and cultured for 3 days in basal medium. After 24, 48, or 72 h, the cells were released from the plates using Accutase and counted in a Bürker chamber. Alternatively, LEC sublines were plated in 96-well plates (3×10^3^ cells/well) and cultured for 3 days in basal or complete medium. The metabolic activity reflecting cell growth and viability was evaluated every day by MTT assay [29]. The values obtained for the wild-type or for the mock-transduced cell line after 24-h culture were taken as 100% in all performed experiments.

### 3D-transmigration assay

The cells of each LEC subline (2×10^5^) were incubated for 16 h in basal medium and then embedded in 150 μl of Matrigel/collagen type I matrix (0.18% collagen type I; Gibco, 10% Matrigel; BD Biosciences) and placed in the 12 mm-diameter Transwell® inserts with a pore size of 8 μm (Merck-Millipore). Basal medium, alone or enriched in VEGF-C (100 ng/ml), was added to the upper and lower chambers of the inserts. After 16 h, cells that had not transmigrated were removed from the upper side of the insert with a cotton swab. The cells that migrated through the membrane were fixed with 2% paraformaldehyde in phosphate-buffered saline (PBS). The membrane was cut out of the insert and mounted in VECTASHIELD mounting medium containing DAPI (Fisher Scientific). Four non-overlapping, randomly chosen fields from each membrane were captured using Zeiss Axiovert 220 fluorescence microscope with Axiocam MRm camera and the cells were counted.

### Flow cytometry

The cells of each LEC subline (1×10^5^) were incubated for 16 h in basal medium, and then stained according to standard procedures for 20 min at 4°C with the following antibodies: (*i*) mouse anti-human integrin α1 (2 μg/ml, Santa Cruz Biotechnology)/FITC-goat anti-mouse IgG (1:100, Jackson ImmunoResearch Lab., Inc.), (*ii*) mouse anti-human integrin α4 (2 μg/ml eBioscience)/FITC-goat anti-mouse IgG (1:100), (*iii*) FITC-mouse anti-human CD31 (1:100, Ancell), or (*iv*) APC-mouse anti-human podoplanin (1:100, BioLegend). Mouse IgG1κ (2 μg/ml, eBioscience) was used as an isotype control for the staining of integrins. Dead cells were excluded from the analysis by 7-aminoactinomycin D (7-AAD) staining. The percentage of the antigen-positive cells and their mean fluorescence intensities were analyzed using FACScalibur Flow Cytometer and Cell-Quest software (Becton Dickinson).

### Cell adhesion assay

The cells of each LEC subline (2×10^4^) were seeded in 96‐well plates coated with fibronectin (20 μg/ml, Merck) or rat tail collagen type I (50 μg/ml, Gibco). The cells were allowed to attach for 15 min to uncoated or fibronectin-coated surfaces, or for 10 min to collagen-coated surfaces. Then the cells that did not adhere to the surface were removed and the wells were rinsed 3 times with PBS. Basal medium containing alamarBlue® was added to the wells and after 3-h incubation under standard cell culture conditions, the fluorescence intensity of the media (a value proportional to the number of adherent cells) was measured at 530/590 nm excitation/emission wavelengths using Synergy H1 Hybrid Reader (BioTek Instruments). In some experiments, the cells were incubated with cyclic RGD peptide (cRGD) prior to the adhesion assay. Briefly, the cells were cultured for 12 h in basal medium, collected with Accutase and incubated in suspension for 45 min under standard conditions to allow recovery of cell surface proteins. Next, the cells were incubated for 30 min in basal medium alone or supplemented with cRGD [cyclo-(RGDfC); tebu-bio] at the concentrations of 1 μM, 10 μM, 100 μM, or 1 mM. Cyclic RGD was present in the medium during the subsequent adhesion assay.

### Time-lapse monitoring of individual cell movements

The cells of each LEC subline (5×10^4^) were seeded in 6-well plates in basal medium and incubated overnight. Then the cells were rinsed with PBS and fresh basal medium was added to the wells. Cell movement was recorded at 37°C and 5% CO_2_ for 6 h at 5-min intervals using Leica DM IRE 2 microscope equipped with FW4000 software. The trajectories of 60 cells per experimental group were analyzed as previously described [30] in order to determine the basic cell motility parameters: the length of cell trajectory (LT), the average speed of cell movement (defined as LT divided by the time of recording), and the net displacement. In experiments testing the influence of growth factor receptor inhibitors on cell movement, the cells (2×10^4^) were seeded in 12-well plates in basal medium and incubated overnight. Then the cells were rinsed with PBS and incubated with fresh basal medium supplemented with: (*i*) EGFR inhibitor (AG-1478, Enzo Life Science; final concentration 1.5 μM), (*ii*) HER2 inhibitor (AG-825, Sigma; final concentration 1 nM), or (*iii*) both inhibitors. The control groups contained DMSO (vehicle), at the corresponding concentrations (0.01 or 0.001%). The recording of cell movement was started 1 h after the exposure of the cells to the inhibitors. Trajectories of 30 individual cells per experimental group were analyzed.

### Analysis of LEC sublines sensitivity to inhibitors of EGFR family members

The cells of each subline (7×10^3^) were seeded in 96‐well plates in basal medium. After 12 h the medium was changed for fresh basal medium supplemented with the inhibitor groups listed above. The cells were incubated for 24 h, after which time the number of viable cells was assessed by alamarBlue staining.

### Angiogenic spheroid assay

Lymphangiogenic sprouting from LEC spheroids was performed according to the procedure described by Korff and Augustin [31] with slight modifications. To generate LEC spheroids, LEC sublines were incubated for 16 h in basal medium, and then transferred to nonadhesive round-bottom 96-well plates (750 cells per well) containing basal medium supplemented with 0.25% (w/v) methylcellulose (Sigma-Aldrich). After 12 h, the spontaneously formed spheroids were harvested, embedded in collagen gel and transferred into prewarmed 24-well plates. The final composition of the gel was: collagen type I (1.5 mg/ml), 0.125% (w/v) methylcellulose, DMEM pH ∼7.4, 15% FCS. After collagen gelation (30 min, 37°C), 200 μl of basal medium (alone or supplemented with 100 ng/ml VEGF-C) was added, and the spheroids were incubated for 24 h under standard conditions. Then the wells were imaged using an inverted microscope (Leica DFC 450C) and the images were analyzed with Image-Pro Plus (Media Cybernetics) to quantify the numbers and lengths of capillary-like sprouts growing from each spheroid. At least ten randomly chosen spheroids per experimental group were subjected to the analysis.

### Human Soluble Receptor Array

Differences between mock-transduced and ADAM17-silenced LEC sublines in the expression and release of relevant proteins were analyzed using Human Soluble Receptor Array (R&D Systems) designed for non-hematopoietic cells. The LEC sublines (1×10^7^ cells each) were plated on six dishes (10-cm-diameter, ~1.6×10^6^ cells per dish in 10 ml of basal medium) and incubated overnight. Then the medium was changed (8 ml per dish) and, after a 16-h incubation, collected and concentrated to a volume of 600 μl, using Amicon Ultra-15 Centrifugal Filter Unit with Ultracel-3 membrane (Millipore). The cells were washed with PBS, collected with Accutase, centrifuged and solubilized for 3 h at 4°C in 600 μl of lysis buffer provided with the array and supplemented with Complete Protease Inhibitor Cocktail (Roche Applied Science). The assay was performed according to the manufacturer's instructions. The membranes were incubated with the buffer containing 300 μl of the concentrated cell media or with 300 μg of the cell lysate proteins. Chemiluminescence signals were captured with Fusion FX5 (Vilber Lourmat) and analyzed with BIO1D software (Scientific Software Group).

### Analysis of integrin expression on LEC surface by Integrin-Mediated Cell Adhesion Array

The expression of individual integrin chains on the surface of LEC was assessed using Alpha/Beta Integrin-Mediated Cell Adhesion Array Kit (Merck-Millipore) according to the manufacturer’s protocol, with slight modifications. Briefly, the cells were detached from the culture plate with Accutase and then incubated in suspension for 45 min to allow for the recovery of cell surface integrins. Then the cells were seeded into the wells (5×10^4^ cells per well) of a 96-well plate coated with antibodies recognizing particular integrins, and incubated for 1 h under standard conditions. Next, the wells were washed three times, and the level of cell binding was estimated by staining with a fluorescent nucleic acid dye CyQuant GR. The fluorescence signal was measured at 485/530 nm excitation/emission wavelengths using Synergy H1 Hybrid Reader (BioTek Instruments).

## Results

### Generation of LEC sublines with silenced expression of ADAM17

**Fig 1:**
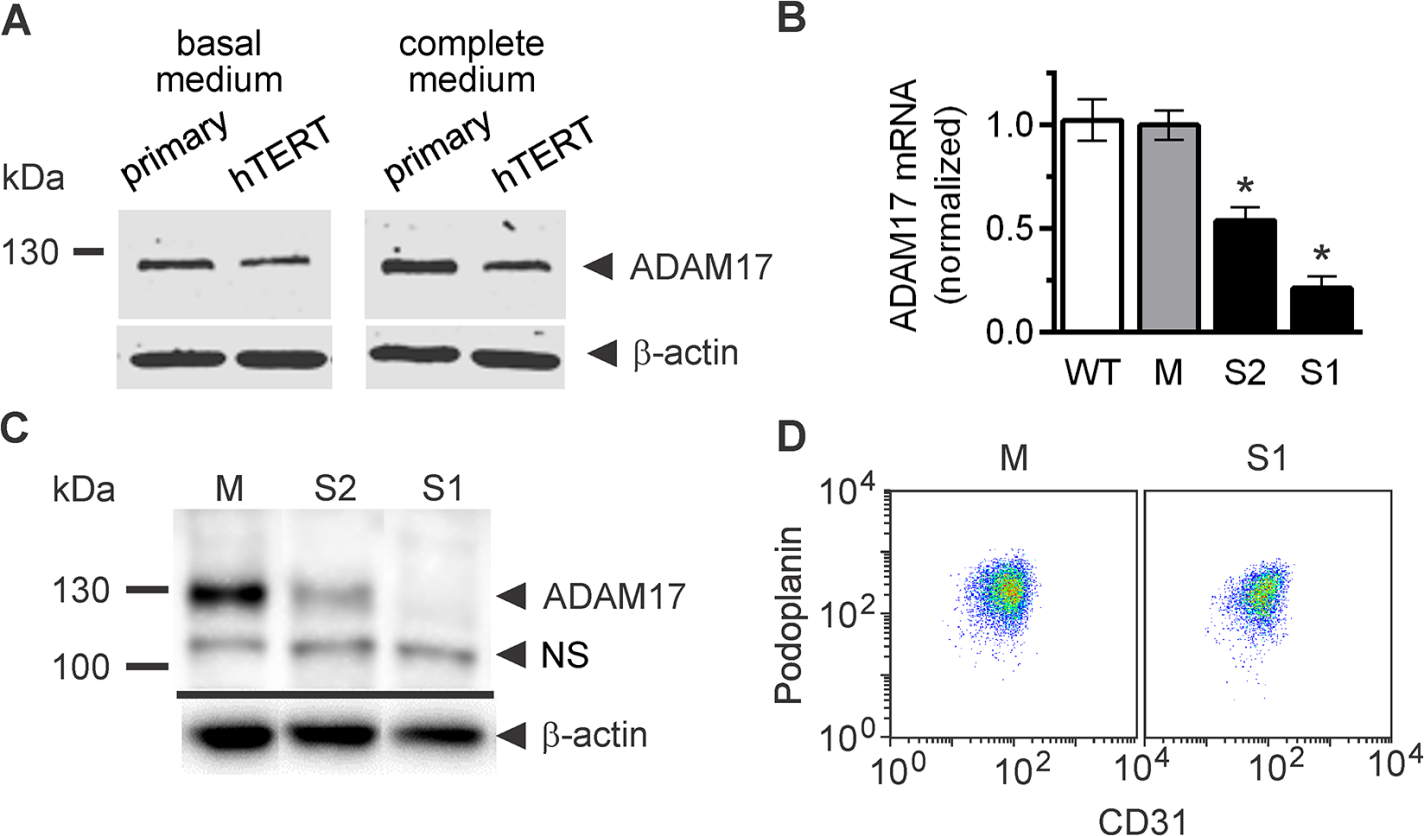
Analysis of ADAM17 silencing in lymphatic endothelial cells (LEC). (**A**) Western blotting analysis comparing the levels of ADAM17 in primary LEC vs. hTERT-immortalized LEC. (**B**) Quantitative RT-PCR analysis of ADAM17 mRNA levels in the wild type LEC (WT), LEC stably transduced with lentiviral vector encoding mock (control) shRNA (M), and LEC stably transduced with lentiviral vectors encoding shRNA sequences targeting ADAM17 –no. 2 alone (S1) or nos. 3 and 5 (S2). Bars represent mean ± SD of three independent experiments performed in duplicates. *P<0.01 *vs* M. (**C**) Western blotting analysis of ADAM17 protein levels in lysates from the LEC sublines. NS–nonspecific band (**D**) Flow cytometry analysis of the LEC markers, CD31 and podoplanin, in M and S1. (**A, B, C**) Shown are representative results of two (**A**) or three (**B, C**) independent analyses performed.

hTERT-immortalized human lymphatic endothelial cells (LEC) produce ADAM17 in similar amounts as primary human dermal microvascular lymphatic endothelial cells (Fig 1A), which makes them a suitable model to study the role of ADAM17 in lymphatic endothelial biology. For this purpose, we generated a LEC subline with stably silenced ADAM17 expression using lentivirus-delivered shRNA targeting ADAM17. Out of five tested shRNA sequences, only one (sequence no. 2) efficiently diminished ADAM17 expression at the mRNA and protein levels (Fig 1B and 1C). The LEC subline (S1) stably expressing this sequence was used in all subsequent experiments. Partial silencing of ADAM17 was achieved by a simultaneous delivery of two shRNA sequences (nos. 3 and 5) (Fig 1B and 1C) and the resulting LEC subline (referred to as S2) was used to exclude possible off-target effects of sequence no. 2. By testing the expression of OAS1, an interferon-inducible gene, we demonstrated that neither the ADAM17-targeting shRNA sequences applied in our study, nor the mock shRNA sequence (further referred to as M) activated the interferon response (data not shown). We also confirmed that the lentiviral transduction and inhibition of ADAM17 synthesis did not affect the lymphatic phenotype of LEC, as they retained the expression of lymphatic markers CD31 and podoplanin (Fig 1D).

### Silencing of ADAM17 does not affect LEC proliferation

**Fig 2:**
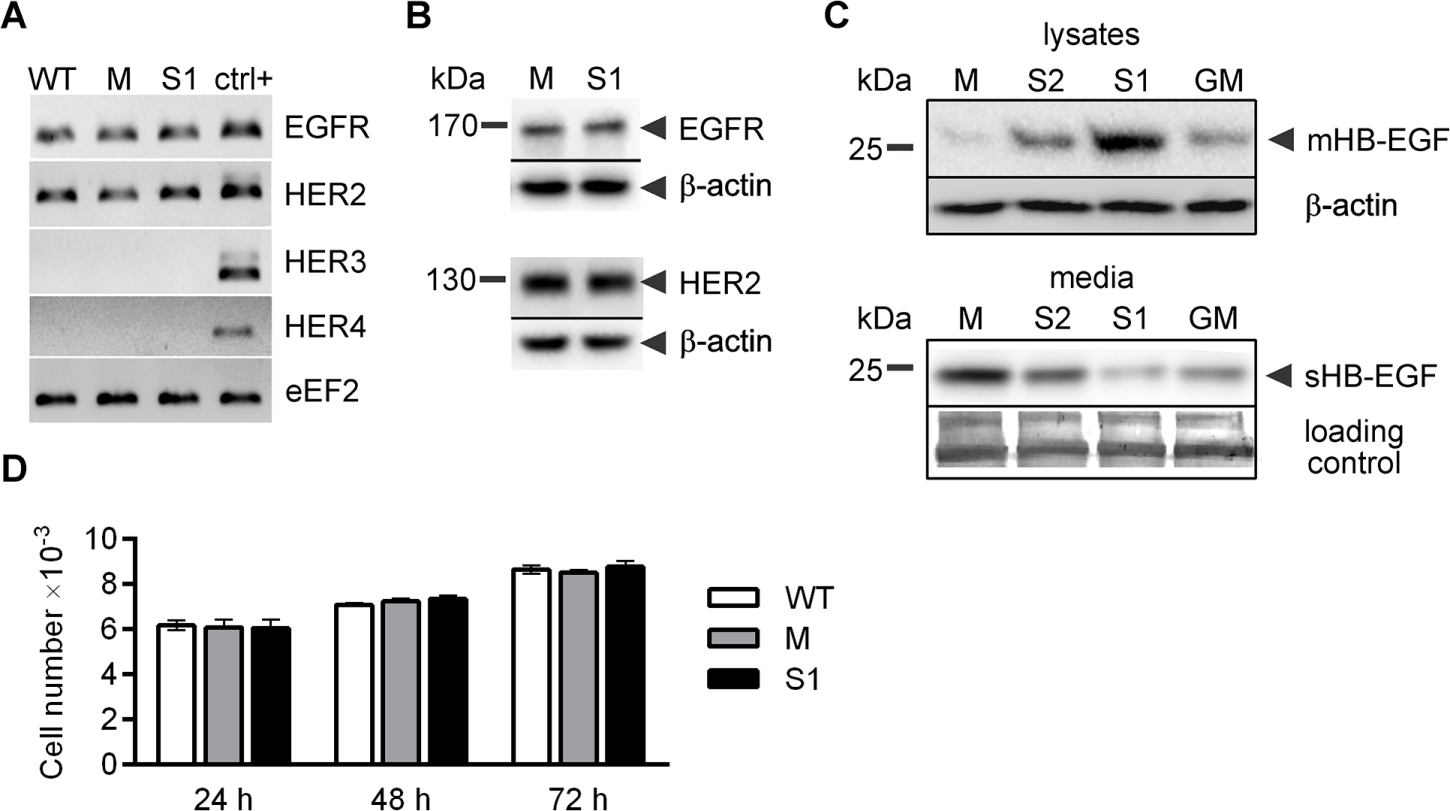
Analysis of the effect of ADAM17 silencing on lymphatic endothelial cells (LEC) proliferation. (**A**) RT-PCR analysis of the expression of members of the EGF receptor family in wild type (WT), M and S1. Positive control (ctrl+)–cDNA from cells that express particular receptors. Reaction mixtures after 40 cycles of quantitative RT-PCR were subjected to electrophoresis in the presence of ethidium bromide (EtBr). The result (shown in photographic negative) is representative of 3 performed experiments. (**B**) Western blotting analysis of EGFR and HER2 in LEC lysates. (**C**) Western blotting analysis of HB-EGF in cell lysates and media of LEC sublines M, S1, S2 and of M exposed for 48 h to 25 μM GM6001 (GM). mHB-EGF, membrane HB-EGF; sHB-EGF, soluble HB-EGF. (**B, C**) β-actin was used as a loading control of lysate proteins; a fragment of blot stained with Coomassie Brilliant Blue after antigen detection procedure was used as a loading control of media proteins. Representative pictures of three independent experiments are shown. (**D**) Changes in the number of WT, M and S1 cultured in basal medium acquired by cell counting. Bars represent mean ± SD of three independent experiments performed in triplicates.

One of the initial steps in the process of new lymphatic vessel formation is the proliferation of LEC. In various *in vitro* models ADAM17 has been shown to potentiate cell proliferation, especially in the case of tumor cells that exhibit autocrine growth stimulation due to the simultaneous expression of EGFR family of growth factor receptors and their ligands. ADAMs-mediated shedding of growth factors strongly facilitates the dimerization or clustering of their receptors and initiation of the signal in the cell. We found that out of four receptors of the EGFR family, LEC express EGFR and HER2 (Fig 2A). Quantitative RT-PCR analysis showed no difference in the expression of *EGFR* and *HER2* between WT, M and S1 (data not shown), confirmed by the equal levels of receptor proteins in the lysates of M and S1 (Fig 2B). We found out that LEC also produce HB-EGF, a substrate of ADAM17, which interacts with both EGFR homodimer and EGFR/HER2 heterodimer. As expected, silencing of ADAM17 resulted in an inhibition of HB-EGF shedding (strong in the case of S1 and moderate in the case of S2), as indicated by the increased levels of HB-EGF in the lysates and decreased levels of the soluble factor in the media of S1 and S2 in comparison to the equivalent measurements obtained for M. GM6001, a broad-spectrum metalloprotease inhibitor also applied in further experiments, had a weaker effect on HB-EGF shedding than ADAM17 silencing in S1 (Fig 2C).

As LEC express both HB-EGF and EGFR family members, we evaluated the impact of ADAM17 silencing on LEC proliferation. To this end, we plated the cells at a low density and directly counted their number after 1, 2, or 3 days of culture in basal medium. LEC proliferated slowly under these conditions; the number of the cells did not increase by more than 45% over the course of 48 h (between 24 h and 72 h of incubation). We did not observe any difference in the cell number between S1 and M at any time point (Fig 2D). The lack of the influence of ADAM17 on LEC proliferation cultured in basal or complete medium was confirmed by MTT assay. The MTT signals indicated that the numbers of both M and S1 in complete medium increased by about 90% over the course of 48 h.

### Silencing of ADAM17 results in increased LEC adhesion and diminished cell motility

**Fig 3:**
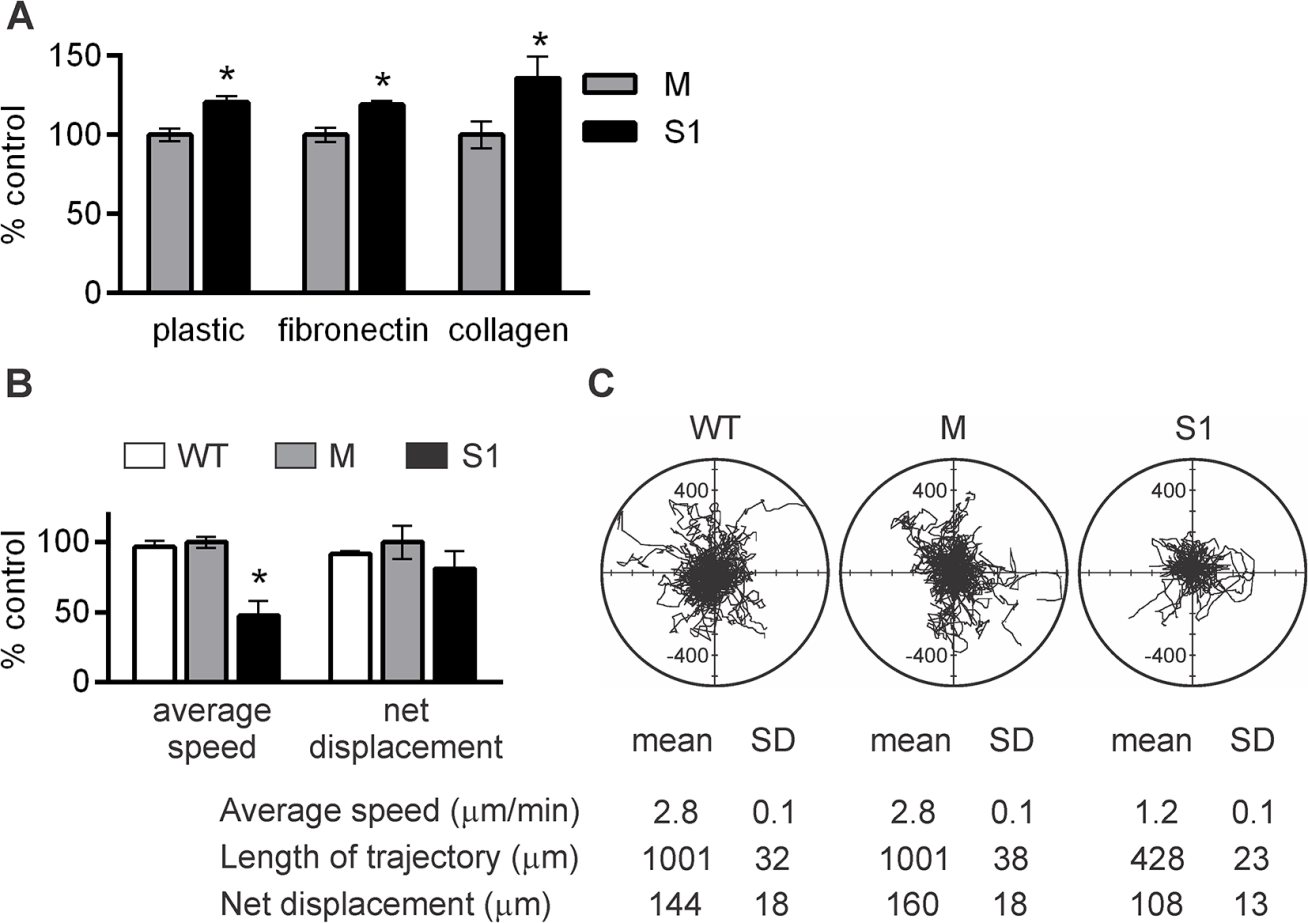
ADAM17 silencing increases adhesion and decreases migration of lymphatic endothelial cells (LEC). (**A**) Adhesion of M and S1 to different substrates assessed by alamarBlue assay. Fluorescence intensity of M that adhered to each substrate was taken as 100%. Bars represent mean ± SD from at least three independent experiments performed in triplicates. *P< 0.05 *vs* M. (**B**) Analysis of single cell motility. Bars represent average values of speed and net displacement of migrating WT, M and S1 from three (M and S1) or two (WT) independent experiments. In each experiment, movements of 60 cells per group were analyzed and normalized to the average values obtained for M. *P<0.01 *vs* M. (**C**) Exemplary trajectories of migrating WT, M and S1 displayed in circular diagrams drawn with the initial point of each trajectory placed at the origin of the plot. Each panel shows the trajectories of 60 cells. The characteristics of cell movement from the representative experiment are presented below the diagram.

Next to proliferation, LEC migration is a key process in lymphangiogenesis. Cell migration requires dynamic interactions between the cells and extracellular matrix (ECM), and there are direct links between cell adhesion and migration. While culturing LEC sublines, we noticed a substantial difference in the speed of cell adhesion between S1 and M. When the suspensions of cells of both sublines were seeded in tissue culture plates, both uncoated and coated with fibronectin or collagen type I, and incubated for a short time period, more S1 attached to the plates in comparison to M (Fig 3A). Thus, silencing of ADAM17 resulted in an increased adhesiveness of LEC to all types of tested surfaces. In contrast, the adhesion rate of S2, in which the expression of ADAM17 was only partially inhibited, was comparable to that of M (data not shown).

To evaluate the possible impact of ADAM17 on LEC motility, we analyzed the movement of WT, M and S1 recorded during a 6-h period (Fig 3B and 3C). The average speeds and hence the length of trajectories of WT and M were the same and more than twice as high as the average speed and the length of trajectory of S1. The differences between the cell lines in the lengths of travelled distances (*i*.*e*. net displacement) were less pronounced because of the randomness of the cell movement (Fig 3B). Partial silencing of ADAM17 also affected LEC motility, although to a lesser extent. The average speed of S2 corresponded to 57% of that of M (data not shown), whereas the average speed of S1 corresponded to 43% of that of M (Fig 3B and 3C). The significant contribution of ADAM17 proteolytic activity to the motility of LEC was further confirmed by the observation that GM6001 decreased the average speed of M to the value observed for untreated S1, while only slightly affecting the movement characteristics of S1 and S2 (S1 Table).

**Fig 4:**
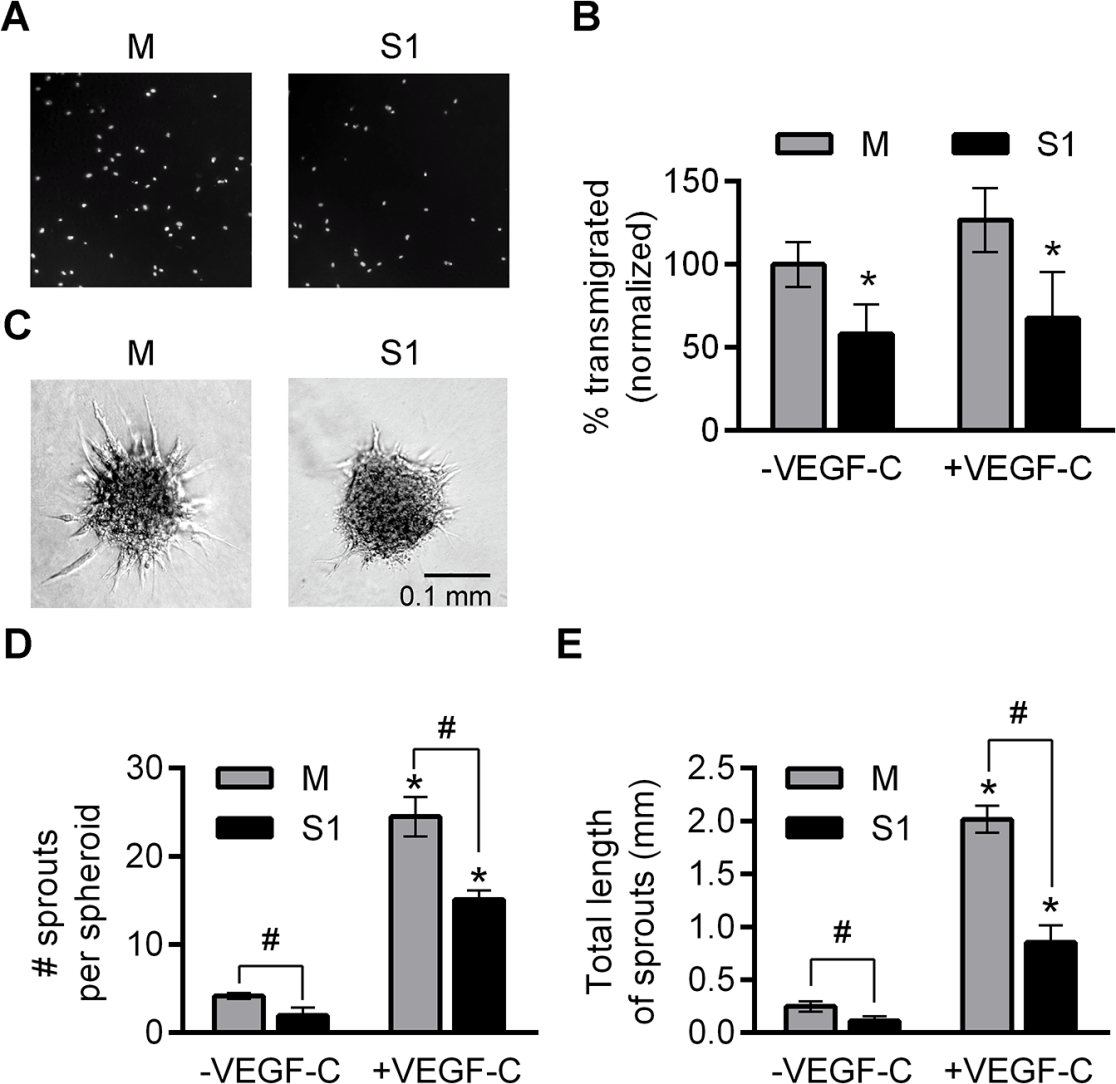
ADAM17 silencing decreases lymphatic endothelial cells (LEC) migration and sprouting. (**A**) Fluorescent images of DAPI-labeled M and S1 that transmigrated through a 3D Matrigel/collagen I matrix. (**B**) Quantification of M and S1 that transmigrated through a Matrigel/collagen I matrix during 16-h incubation in the absence or presence of 100 ng/ml VEGF-C, normalized to untreated M. Bars represent mean ± SD from at least three independent experiments, each analyzed from four microscopic fields per group. *P<0.05 *vs* M, untreated- or treated with VEGF-C, respectively. (**C**) Representative bright field images of sprouting spheroids formed by M and S1 embedded in collagen gel. (**D, E**) Effect of ADAM17 silencing on sprouting of LEC spheroids reflected by the changes in: (**D**) Number of sprouts and (**E**) Total length of sprouts. Bars represent mean ± SD from three independent experiments in which at least 10 spheroids per group were analyzed *P<0.01 *vs* appropriate cell line not treated with VEGF-C; #*P<*0.05 *vs* M, untreated- or treated with VEGF-C, respectively.

Because of the differences between the mechanisms involved in cell migration on flat surfaces (2D) and in 3D surroundings [32], we also compared the migratory potential of S1 and M in a 3D matrix, which is more physiologically relevant for studying cell invasiveness. Compared to M, significantly less S1 transmigrated through the Matrigel/collagen type I matrix over the same period of time. Transmigration of S2 was impaired to the same level as that of S1 (data not shown). The presence of VEGF-C in both compartments of the transwell slightly increased transmigration of all cell lines (Fig 4A and 4B). Unexpectedly, GM6001 did not affect transmigration of either M or S1; however, GW280264X, a specific inhibitor of ADAM10 and ADAM17, strongly inhibited transmigration of M (S1 Fig). These results confirm the importance of ADAM17 in the process of transmigration, at the same time pointing to its complexity. The apparent lack of GM6001 effect might be attributed to its broad target spectrum; elucidation of this phenomenon was beyond the scope of this study.

To confirm the finding that ADAM17 has an impact on the lymphangiogenic potential of LEC, we performed a spheroid sprouting assay, which mimics the first steps of the formation of new capillaries from existing ones [33]. Indeed the number, as well as the total length of sprouts were significantly lower in S1 than in M. The presence of VEGF-C in the matrix significantly stimulated sprouting of both sublines, but the substantial differences between them remained unchanged (Fig 4C–4E). Thus, the less invasive phenotype of S1 was not a result of a diminished sensitivity of the cells to VEGF-C. Both basal and VEGF-C-induced sprouting of M was abolished by GM6001, indicating that the enzymatic activity of metalloproteases plays a crucial role in this process (S2 Fig). Together, these results show that ADAM17 is important for the adhesion properties of LEC, as well as for their motility and invasiveness.

### Signaling through the EGFR family receptors contributes to the motility of LEC

**Fig 5:**
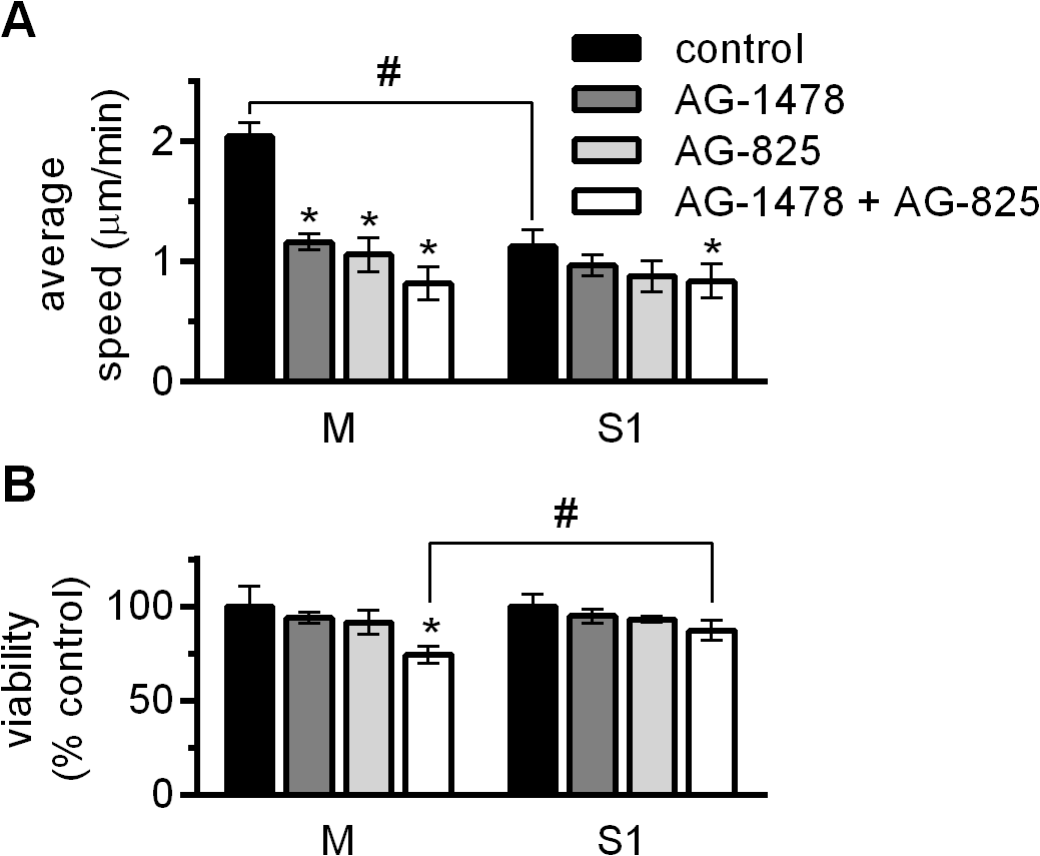
Effect of inhibitors of EGFR family receptors on lymphatic endothelial cells (LEC) motility and viability. (**A**) Analysis of single cell motility. Bars represent average speed ± SD from two independent experiments of migrating M and S1 incubated in the basal medium (control) or in the basal medium supplemented with AG-1478 (inhibitor of EGFR, 1.5 μM), or with AG-825 (inhibitor of HER2, 1 nM), or with the combination of AG-1478 + AG-825. In each experiment, movements of 30 cells per group were analyzed. *P<0.05 *vs* control M or control S1 respectively; #*P<*0.01 *vs* control M. (**B**) Viability of LEC incubated for 24 h in the basal medium alone (control) or in the basal medium supplemented with 1.5 μM AG-1478, or with 1 nM AG-825, or with the combination of AG-1478 + AG-825, assessed by alamarBlue assay. Fluorescence intensity of control M or control S1, respectively, was taken as 100%. Bars represent average values ± SD from six independent experiments performed in sextuplicates. *P<0.05 *vs* control M; #*P<*0.05 *vs* M exposed to both inhibitors.

Most of the effects of ADAM17 on cell motility in various cell lines are attributed to the stimulation of the EGFR family downstream signaling pathways by growth factors released from the plasma membrane as a consequence of the ADAM17 sheddase activity. Since LEC express HB-EGF, the observed differences in motility between M and S1 may result from the differential release of the factor from the plasma membrane of the cells that express and those that do not efficiently express ADAM17. Therefore, we examined to what extent inhibitors of the two receptors of the EGFR family expressed by LEC, EGFR and HER2 will affect their motility. In preliminary experiments, we have established the highest concentrations of the inhibitors that are generally non-toxic to LEC and only slightly affect cell proliferation/viability over the course of 48 h. These concentrations, 1.5 μM for AG-1478, an EGFR inhibitor, and 1 nM for AG-825, a HER2 inhibitor, were used in subsequent experiments. Both compounds strongly inhibited the movement of M, while only weakly affecting S1 motility (Fig 5A). For either cell line, the combination of both inhibitors evoked the strongest effect; the speed of M was decreased by 57% and that of S1 by 23%. It is worth noticing that the motility of both cell types was inhibited to the same absolute value; in the presence of AG-1478 + AG-825 the average speed of M was 0.82 ± 0.14 μm/min and that of S1 was 0.84 ± 0.14 μm/min. Thus, inhibition of the intracellular signal generated by EGFR family receptors abolished the difference in motility between M and S1. The importance of the EGF family/EGFR family axis in LEC biology is also emphasized by the fact that M are more sensitive to the deleterious effects of EGFR- and HER2 inhibitors. For instance, the 24-h incubation with the inhibitors at the concentrations that resulted in the 25% drop in the number of viable M, compared to untreated control, led to a decrease of only 13% in S1 viability (Fig 5B).

### Silencing of ADAM17 affects the levels of several proteins in lysates and media of LEC

To further investigate molecular mechanisms underlying the differences between M and S1, we analyzed cell-associated proteomes and secretomes of both cell lines using Proteome Profiler Array for non-hematopoietic cells. The images of the membranes and complete data concerning all analyzed proteins are included as S1 Data.

**Fig 6:**
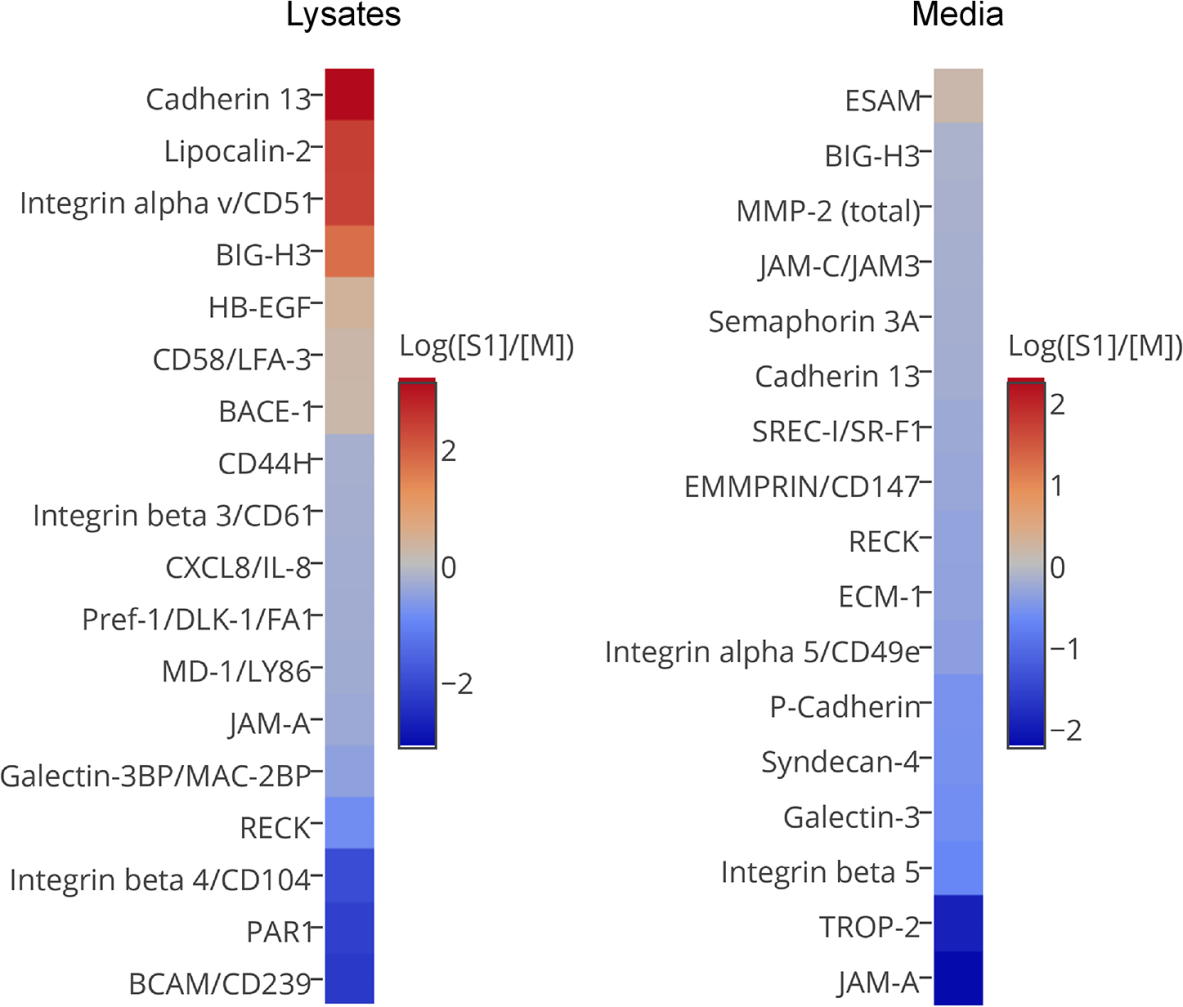
Differences in protein profiles in lysates and media of M and S1 assessed by the human soluble receptor array. Heat map representation of the proteins whose amounts in lysates and/or media differ by at least 30% between M and S1. The fold differences ([S1]/[M]) were calculated as ratios of the chemiluminescent signal volumes. For any given protein, the readout was considered valid when the chemiluminescent signals in technical duplicates did not differ by more than 10%. To facilitate the calculations for limiting cases, *i*.*e*. when a protein was only detected in either M or S1, the mean background chemiluminescence value was taken as a proxy for the lack of signal, and the lower detection limit was set to the lowest value that allowed to distinguish between the background and the signal.

We clearly visualized 60 proteins (out of 119 detectable by the array) and confirmed that the ADAM17 signal in S1 lysate was significantly (by 30%) lower than in M, although the difference did not reflect the 90% silencing of ADAM17 shown in other experiments (Fig 1B and 1C). This result points out that the quantitative data from the analysis of the array require validation in separate experiments. For 19 proteins we observed significant differences between S1 and M in signals either from lysates, or media, or both (Fig 6).

For ADAM17 substrates, higher signals in lysates and lower signals in media are expected to be observed in S1 compared to M. Indeed, higher levels of HB-EGF and TNFR2 (validated ADAM17 substrates) were detected in S1 lysates, and a lower level of syndecan-4, another ADAM17 substrate, was present in the medium of S1 (Fig 6). The prominent differences in the lysate/medium distribution between M and S1 were also observed for other proteins involved in adhesion and migration, such as EMMPRIN-1, cadherin 13 and certain integrin chains (αv, α5, β1, β4). Interestingly, we observed much higher signals in the lysate of S1, compared to M, for two soluble members of the extracellular matrix, BIG-H3 and lipocalin-2. Since this phenomenon cannot be explained by decreased shedding, it is possible that their increased expression in ADAM17-silenced cells is accompanied by their retention in the extracellular matrix fraction that remains associated with the cells during media collection. Alternatively, the expression of these proteins might not differ between S1 and M, but the plasma membrane level of ADAM17 could affect the balance between their ECM retention and release.

The overall results of the array analysis suggest that silencing of ADAM17 may affect the availability of proteins involved in cell adhesion and migration, including BIG-H3 and integrins.

### Silencing of ADAM17 results in increased expression of TGFβ2 and BIG-H3

**Fig 7:**
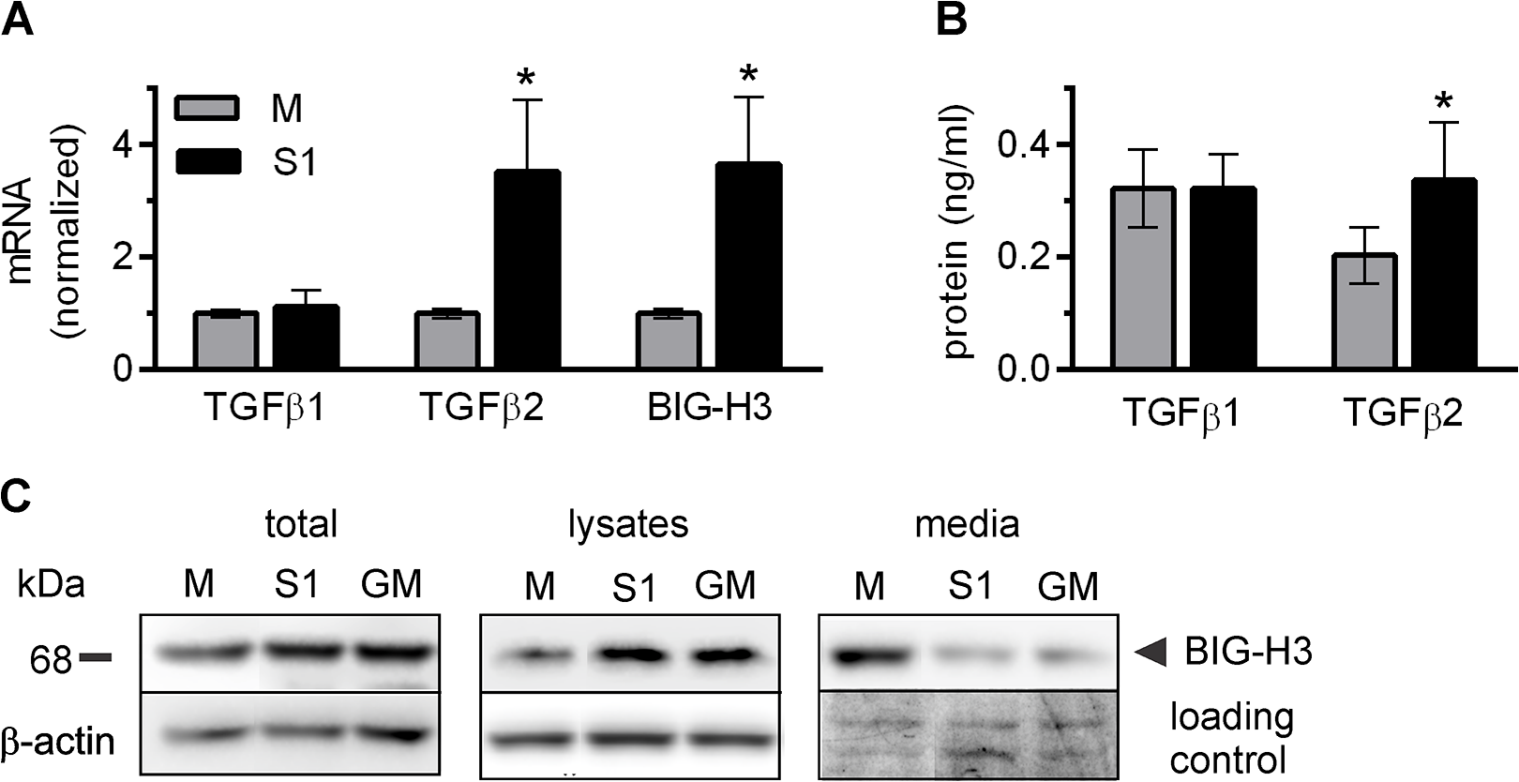
ADAM17 silencing increases the expression of TGFβ2 and BIG-H3 in lymphatic endothelial cells (LEC). (**A**) Quantitative RT-PCR analysis of TGFβ1, TGFβ2 and BIG-H3 mRNA levels in M and S1. Bars represent mean ± SD from five independent experiments performed in duplicates. *P<0.01 *vs* M. (**B**) The levels of TGFβ1 and TGFβ2 released to the medium by M and S1 measured by ELISA. Bars represent mean ± SD from three (TGFβ1) or five (TGFβ2) independent experiments performed in duplicates. *P<0.01 *vs* M. (**C**) Western blotting analysis of the levels of BIG-H3 present in lysates and media of M, S1, and M incubated for 48 h with GM6001 (GM). Total–combined lysates and media. In each lane protein sample from the same number of cells was loaded. A fragment of blot stained with Coomassie Brilliant Blue after antigen detection procedure was used as a loading control of media samples (right lower panel).

An unexpected, prominent difference between S1 and M lysate levels of BIG-H3 (TGF**β**-**I**nduced **G**ene **H3**, also known as TGFβI [**T**ransforming **G**rowth **F**actor **β**-**I**nduced]), an ECM protein involved in cell adhesion and migration, prompted us to investigate how it might be linked to the expression of ADAM17. As BIG-H3 synthesis is strongly stimulated by TGFβ, we analyzed the expression of TGFβ and BIG-H3 in the LEC sublines. As shown in Fig 7A and 7B, the levels of TGFβ1 mRNA, evaluated by qRT-PCR, and of the protein, evaluated by ELISA, did not differ between LEC sublines. In contrast, the levels of TGFβ2 mRNA and protein were significantly higher in S1 than in M. This may explain the greatly enhanced expression of BIG-H3 mRNA in S1 (Fig 7A). In five independent experiments the mRNA levels of both TGFβ2 and BIG-H3 were repeatedly and invariably slightly higher in S2 than in M (by 30% ± 3% for TGFβ2, *P*<0.01 and by 35% ± 4% for BIG-H3, *P<*0.01). Interestingly, the impact of ADAM17 silencing on BIG-H3 cell/medium partitioning resembles that observed for HB-EGF, despite the fact that BIG-H3 is not a membrane protein. Although ADAM17 silencing leads to a total net increase in the level of BIG-H3 (evaluated jointly in media and cell lysates), the protein remains mostly cell-associated, possibly entrapped in ECM. Significantly more BIG-H3 is observed in the lysates and less in the media of S1 when compared with the corresponding fractions of M. The effect of GM6001 on BIG-H3 expression and localization is similar to that of ADAM17 silencing (Fig 7C), suggesting that the proteolytic activity of ADAM17 could be involved in the regulation of BIG-H3 synthesis and trafficking.

### Silencing of ADAM17 affects the surface expression and functions of integrins in LEC

**Fig 8:**
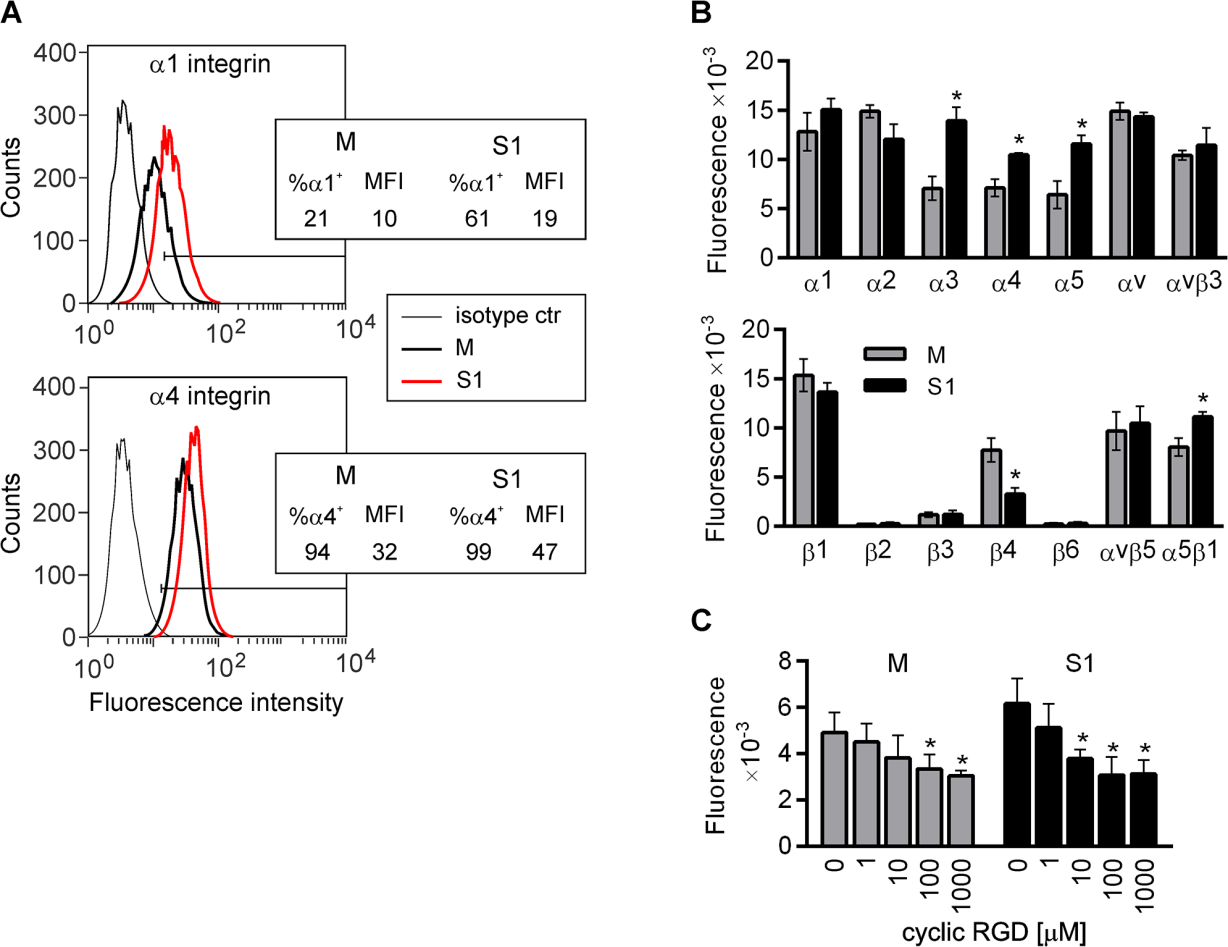
Effect of ADAM17 silencing on surface availability of integrins and their involvement in lymphatic endothelial cell (LEC) adhesion. (**A**) Flow cytometric analysis of α1 and α4 integrin surface expression on M and S1 cultured at the same density. The percentages of α4 integrin-positive cells, as well as the mean fluorescence intensities (MFI, geomean) are presented. A representative result of five independent analyses is shown. (**B**) Availability of particular integrin chains on the cell surface assessed by the level of cell binding to surfaces coated with specific antibodies. Bars represent mean fluorescence signals of CyQuant GR-stained DNA of the cells that interacted with a given antibody ± SD from two independent experiments performed in duplicates. *P<0.05 *vs* M. (**C**) Inhibition of cell adhesion to fibronectin by cyclic RGD peptide analyzed by alamarBlue assay. Bars represent the mean fluorescence signals ± SD from three independent experiments performed in duplicates. *P<0.05 *vs* control M or control S1.

On the basis of the protein array results we hypothesized that the effects of ADAM17 silencing on LEC adhesion and motility may result, at least in part, from the changes in integrin surface expression or availability. Since α1 and α4 integrins were not included in the array, but have been shown to play a role in pathological lymphangiogenesis [34], we analyzed their levels on the surface of M and S1 by flow cytometry. As shown in Fig 8A, the surface expression of both α1 and α4 integrins was higher in S1 than in M. The mean fluorescence intensity (MFI) of S1 stained with α4-specific antibody was higher than the MFI of M in a range of 135–210% (average = 160%; SD = 31%; *P*<0.05), in 5 experiments. LEC displayed low α1-specific fluorescence. Only between 21 and 55% (average = 36%; SD = 14%; n = 4) of M were considered as α1-positive with the MFI of ungated population ranging from 9.5 to 19.2 (average = 13.3; SD = 4.2). The percentage of α1-positive S1 was twice as high as that of M (average = 68.3%; SD = 5.5%; *P*<0.05) and the MFI of ungated cells was also higher (average = 20.4; SD = 3.5). Interestingly, staining of S2 with α1-specific antibody was more pronounced than that of M and weaker than that of S1 (average = 47% positive cells, SD = 2.8% and MFI = 15, SD = 2.8) in 2 independent experiments (data not shown).

To validate the differences in the integrins levels observed by the protein array and flow cytometry, we analyzed the interactions of S1 and M with a set of antibodies directed towards particular α and β chains of integrins. As shown in Fig 8B, significantly more S1 than M interacted with surfaces coated with antibodies recognizing α3, α4, and α5 chains. The importance of increased availability of α5 on the surface of S1 was confirmed by the higher level of binding of S1 to the antibody recognizing the complex of α5 with its sole β chain partner, α5β1. S1 also interacted with an anti-α1 antibody slightly more efficiently than M, but the difference between the cell lines did not reach statistical significance. Interestingly, the decreased level of β4 in S1 lysates demonstrated by the protein array (Fig 6 and S1 Data) was in concordance with the diminished interaction of S1 with an anti-β4 antibody, in comparison to M (Fig 8B). The possible significance of this finding is at the moment difficult to interpret, as we did not analyze in detail the expression of α6, the sole partner of β4. In a single experiment, the levels of S2 binding to antibodies recognizing particular integrin chains resembled these of M rather than S1, with the exception of the interaction between S2 with the anti-α5β1 antibody. The signal of S2 bound to anti-α5β1 exceeded this of M by more than 60%.

To verify the possible functional significance of the increased levels of certain integrins on S1, we analyzed the effect of the inhibition of interaction between integrins and fibronectin on LEC adhesion using a cyclic RGD peptide (cRGD), which mimics the integrin recognition site within many ECM proteins, including fibronectin. As shown in Fig 8C, cRGD inhibits the early adhesion of both M and S1 to fibronectin-coated surface in a concentration-dependent manner. Significant inhibition was observed for a 10-μM (for S1) and 100-μM (for M) concentration of the compound; however, the extent of inhibition of S1 adhesion was significantly higher than that of M (50% and 32% of inhibition, respectively). In other words, cRGD abolished the difference in the speed of cell adhesion to fibronectin between the two LEC sublines, as in the presence of cRGD the same numbers of S1 and M adhered to fibronectin within the same period of time. Thus, ADAM17 may influence LEC biology also by affecting integrin surface expression and/or functionality.

## Discussion

Overexpression of ADAM17, accompanied by an increased shedding of numerous growth factors, cytokines, and cell adhesion molecules, has been widely linked to the progression of cancer and the promotion of inflammation [23]. This view has recently been challenged by Yoda et al., who showed that the levels of validated ADAM17 substrates were not increased in the serum of ADAM17-overexpressing transgenic mice compared to the wild-type animals, undermining the correlation between ADAM17 expression and its activity [35]. Nevertheless, systemic overexpression of ADAM17 may have pathological consequences despite the lack of correlation between the level of ADAM17 expression and the plasma levels of its substrates [36]. Furthermore, tissue-specific deletion or hypomorphic knock-in of *Adam17* leads to a decrease in the serum levels of ADAM17 substrates [37, 38]. The results of numerous *in vivo* studies using these models strongly support the notion that ADAM17 expression level is important in inflammation-related processes [11, 39]. Increased expression of ADAM17 is not a trigger of pathologies, but rather allows for the full manifestation of the activity of ADAM17 substrates whose expression is stimulated during immune activation or cancer-related processes. Therefore, silencing of ADAM17 is a useful methodology to study the significance of ADAM17 in various cells and processes. We used this approach to investigate how ADAM17 impacts lymphangiogenesis. To our knowledge, this is the first *in vitro* study of the role of ADAM17 in LEC biology.

Our data indicate that ADAM17 has a significant contribution to LEC migration and sprouting, but not proliferation. ADAM17 has been shown to promote migration of tumor cells [14, 40], non-transformed keratinocytes, and vascular endothelial cells [21]. Most reports demonstrating ADAM17-mediated stimulation of cell migration pointed to growth factor-dependent mechanisms [14, 21, 40]. For example, the increased migration of human umbilical vein endothelial cells (HUVEC) in response to VEGF-A involves activation of ADAM17, which releases HB-EGF from the plasma membrane, resulting in the stimulation of EGFR [21]. The importance of ADAM17-activated HB-EGF-EGFR/HER2 signaling axis for LEC motility is highly probable, as specific inhibitors of these receptors abolished the difference in migration rates between ADAM17-proficient and ADAM17-silenced LEC.

Our results also suggest that ADAM17 may influence LEC adhesion and migration by modulating the availability or functionality of integrins. We show a higher reactivity of S1, compared to M, with antibodies recognizing the α1, α3, α4, α5 integrin chains, and the α5β1 integrin. We have excluded the possibility that ADAM17 affects the expression of these integrins by qRT-PCR analysis (data not shown). However, ADAM17 could influence integrin availability/activity in multiple other ways. ADAM17 contains a disintegrin domain which lost the RGD sequence during evolution but retained the ability to bind to α5β1 and α6β1 integrins [8, 41]. Thus, interactions of ADAM17 with α5β1 and α6β1 (in *cis*) could diminish the availability of these integrins for interactions with the ECM. It is also possible that the interplay and cross-talks between validated ADAM17 substrates and integrins may affect integrin-mediated adhesion and migration [42–44]. Another conceivable scenario involves potential cleavage of certain integrins by ADAM17. Although integrins are considered to be ADAMs interacting partners rather than their substrates, there are reports demonstrating ADAM17-dependent shedding of β1D and Mac-1 [45, 46]. Whether other integrins are substrates of ADAM17 remains to be determined.

Remarkably, our results point to a potent impact of ADAM17 on LEC ECM protein profile. Silencing of ADAM17 not only leads to the increased expression of BIG-H3, but also significantly affects its distribution, increasing the cell-associated BIG-H3 pool. BIG-H3 is believed to function as an adaptor in the interactions between integrins and the ECM components including collagens, fibronectin, and laminins; and its effects on various cells are very diverse [47–50]. Interestingly, Irigoyen *et al*. found that BIG-H3 was responsible for the hypoxia-induced increase in adhesion of primary human dermal LEC to extracellular matrix components such as collagen type I and IV, and fibronectin [51]. It is possible that, in the case of ADAM17-silenced LEC, the increased level of ECM BIG-H3 also contributed to their increased adhesion and impaired migratory potential.

Interestingly, silencing of ADAM17 results in an increased transcription of TGFβ2, without changing the transcription of either TGFβ1 nor that of TGFβ3 (data not shown). Differential regulation of the expression of these isoforms is possible as *e*.*g*. human papillomavirus type 16 and 17 proteins were shown to inhibit the differentiation-dependent expression of TGFβ2, but did not alter the expression of TGFβ1 or 3 in cervical keratinocytes [52]. Although the various TGFβ isoforms interact with the same major receptor complex, TGFβ-RI (ALK5)-RII, their effects may vary, as TGFβ2 does not bind to endoglin and therefore cannot activate ALK1 and Smad1/5/8 [53]. It is worth noting that, out of the three mammalian TGFβ proteins, TGFβ2 was found to be the most important isoform in the lymphatic network morphogenesis during mouse development [54].

The studies that addressed the question of the role of TGFβ in pathological lymphangiogenesis indicated that TGFβ inhibits this process [55–59]. Our results are in agreement with this notion, as silencing of ADAM17 resulted in an increase in TGFβ2 expression in parallel with inhibition of the invasive behavior of LEC. Although in most of the studies cited above, the TGFβ1 isoform was investigated, the extension of the general conclusion to all isoforms seems to be justifiable because inhibition of the common TGFβ receptor [58] leads to the same effects as the inhibition of TGFβ1 activity.

Our finding that silencing of ADAM17 may affect TGFβ expression adds another level of complexity to the multifaceted relationship between these two molecules. ADAM17 is known to down-regulate TGFβ signaling by shedding the extracellular domain of TGFβ-RI [60], as well as that of vasorin, a protein that, in its soluble form, binds to and neutralizes TGFβ [61]. Thus, it is possible that silencing of ADAM17 in LEC not only increases the levels of TGFβ2, but also facilitates the initialization of TGFβ-signaling by increasing the receptor availability and decreasing the level of soluble TGFβ-neutralizing factor. On the other hand, TGFβ promotes ADAM17 activation and shedding of HB-EGF, and possibly other growth factors [62–64], which explains the observed activation of EGFR signaling in response to TGFβ. It seems that the net effect of TGFβ on a certain biological process may result from the cross-talk between TGFβR- and EGFR-mediated signals.

In conclusion, the activity of ADAM17 influences the transcriptome and the proteome/secretome of lymphatic endothelial cells. Impaired EGFR signaling, increased expression of TGFβ2 and BIG-H3, and the altered surface availability/functionality of integrins and cell adhesion molecules in ADAM17-silenced cells may account for their significantly lower migratory and invasive potential, compared to ADAM17-proficient LEC. As ADAM17 is often overexpressed in tumors and promotes their progression, it has been recognized as a promising target for cancer therapy. A number of ADAM17 specific inhibitors, both small-molecule compounds [65] and monoclonal antibody-based drugs [66], are currently under development. Our results indicate that such therapeutics might not only directly inhibit tumor growth, but could also affect tumor growth and spreading by reducing the tumor lymphangiogenesis.

## Supporting Information

S1 Table Influence of a broad range metalloprotease inhibitor GM6001 on LEC motility. (DOCX)

S1 Fig Influence of GM6001, a broad range metalloprotease inhibitor, and GW280264X, ADAM10- and ADAM17-specific inhibitor, on LEC transmigration through Matrigel/collagen type I matrix. (DOCX)

S2 Fig Influence of a broad range metalloprotease inhibitor GM6001 on LEC sprouting. (DOCX)

S1 Data The results of Proteome Profiler Array–Human Soluble Receptor Array Non-hematopoietic panel. Complete data table and images of the array membranes.
(DOCX)
