# Supplementary material for: ADAM17 Promotes Motility, Invasion, and Sprouting of Lymphatic Endothelial Cells
Source: PLoS One. 2015 Jul 15;10(7):e0132661. doi: 10.1371/journal.pone.0132661 (PMC4503755; doi:10.1371/journal.pone.0132661)
Supplement: S1 Table — (DOCX) [file pone.0132661.s001.docx]

S1

Influence of a broad range metalloprotease inhibitor GM6001 on LEC motility.

The cells of each LEC subline (5×10^4^) were seeded in 6-well plates in basal medium and incubated overnight. Then the cells were rinsed with PBS and fresh basal medium containing GM6001 at an indicated concentration or appropriate volume of DMSO (GM6001 solvent) was added to the wells. Cell movement was recorded at 37°C and 5% CO_2_ for 6 h in 5-min intervals using Leica DM IRE 2 microscope equipped with FW4000 software. The movement characteristics of 30 cells per experimental group were analyzed.

|  | **M** | | | **S1** | | | **S2** | | |
| --- | --- | --- | --- | --- | --- | --- | --- | --- | --- |
|  | ctrl | GM6001 (µM) | | ctrl | GM6001 (µM) | | ctrl | GM6001 (µM) | |
|  |  | 12.5 | 25 |  | 12.5 | 25 |  | 12.5 | 25 |
| Average speed | 2.6 (0.1) | 1.7  (0.1) | 1.5  (0.1) | 1.4  (0.1) | 1.3  (0.1) | 1.2  (0.2) | 1.6  (0.1) | 1.3  (0.2) | 1.4  (0.1) |
| Average distance | 935  (49) | 611 (61) | 532 (33) | 493 (65) | 475 (54) | 425 (47) | 569 (62) | 464 (44) | 488 (58) |
| Net displacement | 246 (22) | 221 (39) | 141 (46) | 179 (52) | 182 (80) | 160 (36) | 205 (46) | 157 (41) | 152 (57) |

Conclusion: GM6001 strongly inhibits individual cell movement of M and negligibly that of S1 and S2.
