# Supplementary material for: ADAM17 Promotes Motility, Invasion, and Sprouting of Lymphatic Endothelial Cells
Source: PLoS One. 2015 Jul 15;10(7):e0132661. doi: 10.1371/journal.pone.0132661 (PMC4503755; doi:10.1371/journal.pone.0132661)
Supplement: S1 Fig — (DOCX) [file pone.0132661.s002.docx]

S1 Fig.

Influence of GM6001, a broad range metalloprotease inhibitor, and GW280264X, ADAM10- and ADAM17-specific inhibitor, on LEC transmigration through Matrigel/collagen type I matrix.

M and S1 cells (2×10^5^) were incubated for 24 h in basal medium alone or supplemented with GM6001 (25 µM), GW280264X (10 µM), or in DMSO (inhibitors’ solvent) at an appropriate concentration. Then the cells were embedded in 150 µl of Matrigel/collagen type I matrix and placed in 12 mm-diameter Transwell® inserts (8 µm pore-size). Basal medium, alone or supplemented with GM6001, or GW280264X, or DMSO was added to the upper and lower chambers of the inserts. After 16 h, the cells that migrated through the membrane were fixed with 2% paraformaldehyde in phosphate-buffered saline (PBS). The membrane was cut out of the insert and mounted in VECTASHIELD mounting medium containing DAPI. Four non-overlapping randomly chosen fields from each membrane were captured using a Zeiss Axiovert 220 fluorescence microscope with an Axiocam MRm camera and the cells were counted.

Conclusion: GM6001 does not influence transmigration of either M or S1 through the Matrigel/collagen type I matrix. Conversly, the specific inhibitor of ADAM10 and ADAM17 strongly inhibits transmigration of M. Bars represent means ± SD from 4 (GM6001) and 2 (GW280264X) independent experiments. **P<*0.05 *vs* control (DMSO).
