# Supplementary material for: ADAM17 Promotes Motility, Invasion, and Sprouting of Lymphatic Endothelial Cells
Source: PLoS One. 2015 Jul 15;10(7):e0132661. doi: 10.1371/journal.pone.0132661 (PMC4503755; doi:10.1371/journal.pone.0132661)
Supplement: S2 Fig — (DOCX) [file pone.0132661.s003.docx]

S2 Fig.

Influence of a broad range metalloprotease inhibitor GM6001 on LEC sprouting.

M cells were incubated for 24 h in basal medium and then transferred to nonadhesive round-bottom 96-well plates (750 cells per well) containing basal medium supplemented with 0.25% (w/v) methylcellulose. After 24 h, the spontaneously formed spheroids were harvested, embedded in collagen gel and incubated in 24-well plates for 24 h in basal medium alone or supplemented with 100 ng/ml VEGF-C. GM6001 (25 µM) or DMSO (vehicle) was present in the medium throughout the entire procedure. Microscopic images (Leica DFC 450C) were analyzed with Image-Pro Plus (Media Cybernetics) to quantify the numbers and lengths of capillary-like sprouts growing from each spheroid. At least ten randomly chosen spheroids per experimental group were subjected to the analysis.





Conclusion: GM6001 strongly inhibits basal and VEGF-C-induced sprouting of M cells.
