# Supplementary material for: ADAM17 Promotes Motility, Invasion, and Sprouting of Lymphatic Endothelial Cells
Source: PLoS One. 2015 Jul 15;10(7):e0132661. doi: 10.1371/journal.pone.0132661 (PMC4503755; doi:10.1371/journal.pone.0132661)

S1 Data

The results of Proteome Profiler Array – Human Soluble Receptor Array

Non-hematopoietic panel

The following proteins were detected neither in the lysates, nor in the media of LEC:

ADAM8, ADAM10, Amphiregulin, APP, Cadherin-4, -11, E-, VE-cadherins, CD9, CD23/FcεRII, CD40, CD90/Thy1, CEACAM-1/CD66a, CEACAM-5/CD66e, CHL-1/L1CAM-2, COMP/Thrombospondin-5, CXCL1/Fractalkine, Desmoglein 2, Erb1 - 4, Endoglycan, EpCAM/TROP-1, Epiregulin, E-selectin, Galectin-2, HPRG, Integrins: α3, α6, α9, β6, Jagged-1, JAM-B, LOX-1, LRP-6, MEPE, MUCDHL, NCAM-1, NCAM-L1, Nectin-4, Neurotrimin, Notch-1, NrCAM, Osteopontin, Periostin/OSF-2, Podocalyxin, SREC II, Stabilin-1, Syndecan-1/CD138, Thrombin, Thrombospondin-2, TIMP-3, VAP-1/AOC3, VCAM-1, VEGFR1/Flt-1. ACE and IL1-RII were not detected in LEC media and their signals in lysates of both M and S1 were at the detection limit. N-Cadherin and Cadherin-4/R-Cadherin were not detected in LEC lysates and the signals in media of both M and S1 were at the detection limit.

| Protein | Chemiluminescence 10^-3^ | | | |
| --- | --- | --- | --- | --- |
|  | **Lysates** | | **Media**^1^ | |
|  | **M** | **S1** | **M** | **S1** |
| ADAM15 | 662 | 623 | NS^2^ | NS |
| ADAM9 | 1593 | 1434 | 351 | 311 |
| ALCAM | 557 | 456 | 298 | 273 |
| BACE-1 | 130 | 220 | NS | NS |
| BCAM/CD239 | 62 | NS | NS | NS |
| BIG-H3 | **5** | **270** | 1188 | 830 |
| C1q R1/CD93 | 1265 | 1353 | NR^3^ | 1275 |
| Cathepsin D | 1654 | 1792 | 1277 | NR |
| Cadherin 13 | NS | **406** | 262 | 160 |
| P-Cadherin | NS | NS | **109** | **30** |
| CD155 | 202 | NR | NS | NS |
| CD31/PECAM-1 | 965 | 867 | 363 | 336 |
| CD36/SR-B3 | 278 | 356 | NS | NS |
| CD40 Ligand | 217 | 189 | NS | NS |
| CD44H | 110 | 60 | NS | NS |
| CD58/LFA-3 | 81 | 140 | NS | NS |
| CD99 | 544 | 577 | NS | NS |
| Clusterin | NS | NS | 276 | 269 |
| CRELD2 | 2889 | 3095 | 291 | NR |
| CXCL8/IL-8 | **857** | **421** | NR | NR |
| ECM-1 | 473 | 410 | **1488** | **648** |
| EMMPRIN/CD147 | 2813 | 3106 | **809** | **402** |
| Endoglin/CD105 | 723 | 653 | 297 | 246 |
| ESAM | 2293 | 1760 | 1208 | 1720 |
| Galectin-1 | 334 | 241 | NS^2^ | NS |
| Galectin-3 | 1653 | 1777 | **338** | **83** |

| Protein | Chemiluminescence 10^-3^ | | | |
| --- | --- | --- | --- | --- |
|  | **Lysates** | | **Media** | |
|  | **M** | **S1** | **M** | **S1** |
| Galectin-3BP/MAC-2BP | **112** | **33** | 313 | 248 |
| HB-EGF | **29** | **70** | NS | NS |
| ICAM-2/CD102 | DL^4^ | DL | 382 | 301 |
| IL-15 R alpha | 338 | 296 | NS | NS |
| Integrin alpha v/CD51 | **2** | **455** | NS | NS |
| Integrin alpha 5/CD49e | 658 | 735 | **205** | **81** |
| Integrin beta 1/CD29 | 2621 | 3406 | 473 | 402 |
| Integrin beta 2/CD18 | 135 | 156 | DL | DL |
| Integrin beta 3/CD61 | 100 | 52 | NS | NS |
| Integrin beta 4/CD104 | **100** | DL | NS | NS |
| Integrin beta 5 | 1976 | 1698 | **263** | **53** |
| JAM-A | **509** | **211** | **174** | DL |
| JAM-C/JAM3 | 1066 | 1057 | 387 | 248 |
| Lipocalin-2 | NS | **75** | NS | NS |
| MCAM/CD146 | 701 | 638 | 212 | 210 |
| MD-1/LY86 | **188** | **87** | NS | NS |
| MMP-2 (total) | DL | DL | 279 | 187 |
| Nectin-2/CD112 | 1029 | 1202 | 672 | NR |
| PAR1 | 156 | DL | NS | NS |
| Pref-1/DLK-1/FA1 | **148** | **72** | NS | NS |
| RECK | **73** | **10** | 156 | 71 |
| Semaphorin 3A | NS | NS | 74 | 47 |
| SREC-I/SR-F1 | 1322 | 1162 | 206 | 111 |
| Stanniocalcin 1 | NS | NS | 112 | 87 |
| Syndecan-4 | NS | NS | **141** | **38** |
| TACE/ADAM17 | 336 | 238 | NS | NS |
| Thrombospondin-1 | 1014 | 967 | 436 | 491 |
| TIMP-1 | 463 | 390 | 926 | NR |
| TIMP-2 | NS | NS | 630 | 698 |
| TIMP-4 | NS | NS | 183 | 157 |
| TNFR2 | 47 | 48 | NS | NS |
| TROP-2 | 499 | 432 | **90** | DL |
| VEGFR2 | NS | NS | 106 | 104 |

^1^Signals from media and lysates should not be compared with each other. The samples of lysates were from around 2.5 times less cells than the samples of media. ^2^NS – no signal. ^3^NR – not reliable, the differences between signal volumes of spots in duplicates exceeded 10%. ^4^DL – signal at the detection limit. In the cases where differences between signal volumes ≥50% the numbers are in bold.


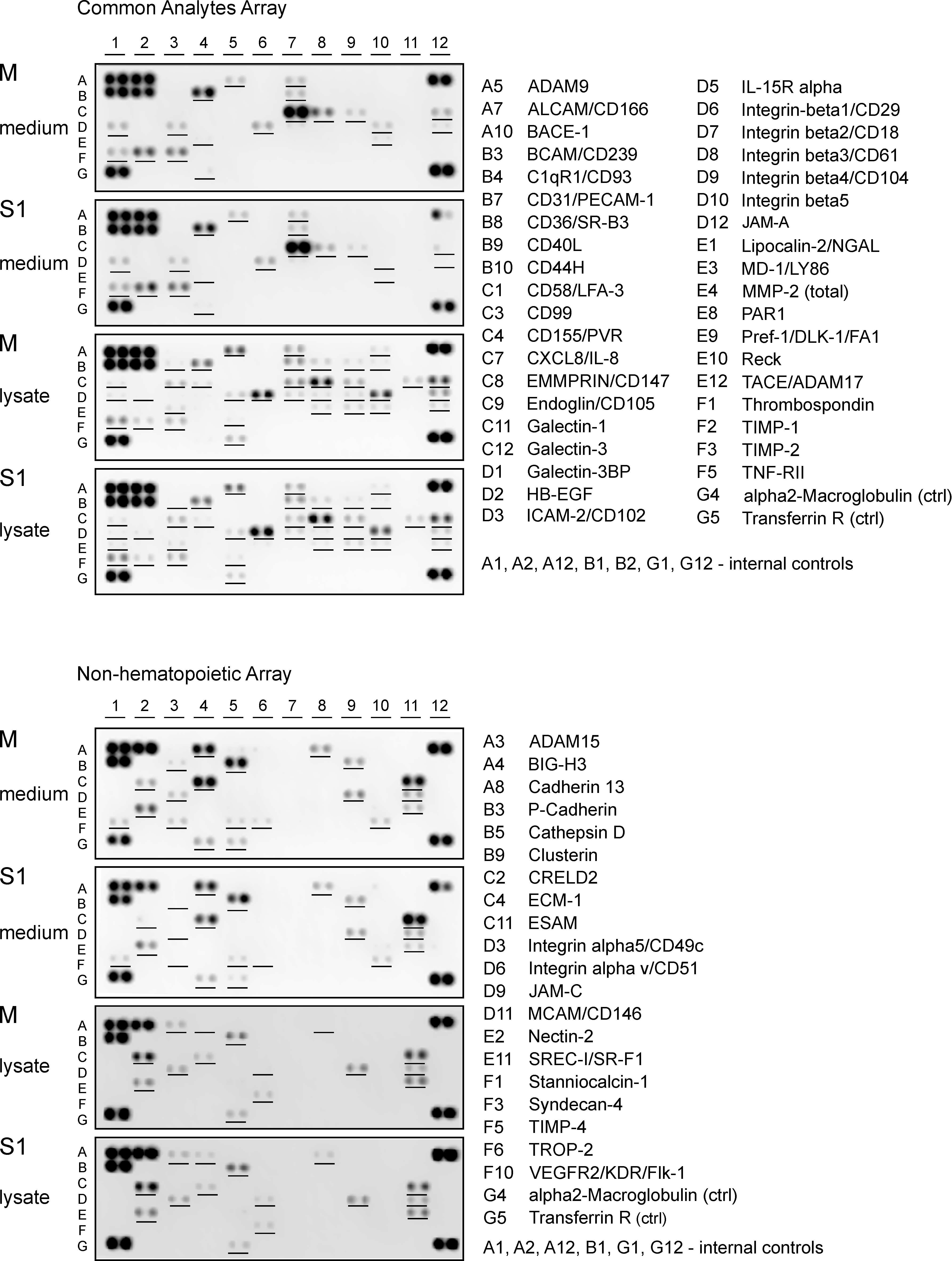

Supplement: S1 Data — Complete data table and images of the array membranes. (DOCX) [file pone.0132661.s004.docx]
